# Case of a Woman Who Underwent the Section of the Symphysis Pubis

**Published:** 1790

**Authors:** John Welchman

**Affiliations:** Surgeon at Kington in Warwickshire


VI. *Cafe of a Woman who underwent the Section of the Symphyfis Pubis. By Mr.* John Welchman, *Surgeon at Kington in Warwickfhire.*

MARY ORDWAY, the patient whofe cafe I am about to relate, was perhaps as unpromifing a fubject for any operation as could have offered. About the year 1774, when big with her fecond child, fhe fell down, as fhe was carrying a yoke of water, and ftruck the fmall of her back againft the edge of a ftone ftep. She complained of great pain at the time; but, upon lofing fome blood, grew better, and had a natural eafy labour. Two years after, I delivered her of another child after three days fevere labour. She then told me fhe had been troubled with rheumatic pains, and had been very lame ever fince her laft lying in, which fhe thought was owing to taking cold. I ordered her medicines which relieved her, her, and heard no more of her till I was called on Monday, September 2d, 1782. She was now in her thirty-ninth year, and her laſt child was ſix years old. I was ſhocked at her preſent appearance; for, from having been a ſtout woman, of about five feet ſix inches high, ſhe was now reduced to leſs than four feet in height; her knees and chin almoſt meeting, and the muſcles being ſo contracted, that her knees could be ſeparated but very little without occaſioning great pain. I learnt, upon inquiry, that ſhe had not been able to walk, without two ſticks, for a long time; and that for the laſt two years ſhe had moſtly been confined to her elbow chair or her bed. Upon examining *per vaginam* I was ſtill more diſtreſſed. I found the tuberoſities of the iſchia ſo near together, that I could but juſt get my fore finger betwixt them; and, notwithſtanding this impediment, I could reach quite round the jetting in of the os ſacrum.

The child lay very high, the nates preſenting, and her pains were moderate. She had been feveriſh ſeveral days; her pulſe was at 100; ſhe took ſcarcely any nouriſhment, vomited frequently, and got no ſleep. I directed a ſaline mixture, with anodynes, to be adminiſtered niftered occafionally. Thefe relieved her fick-nefs, but procured very little reft.

On Tuefday morning (Sept. 3) a flight flooding came on, and the weather at this time being unufually warm, the door and window were kept open, and fhe drank freely of tincture of rofes. By thefe methods her flooding was fuppreffed, and her pains became ftrong, but the child did not advance; her pulfe was now at 110, but weak. During this day and night my fon frequently vifited her with me, and from every circumftance we were both fully convinced that fhe had not the leaft chance of living without an operation.

Early on Wednefday morning I propofed the fection of the pubis, to which fhe readily confented; and I was the more inclined to perform it, as fhe conftantly affirmed fhe felt the child move. But as the operation had never been performed in England upon a living fubject, we were defirous of having the fanction of fome perfon of experience to the neceffity of the operation, that we might not be accufed of rafhnefs in cafe the patient fhould not recover, and of which indeed there was but little hope. I therefore difpatched a meffenger to Warwick for Dr. Haddow; but he was unfortunately engaged, engaged, and informed me, that he could not be with me till the next morning.

The flooding had now returned, and no time was to be loſt; I therefore, with the aſſiſtance of my ſon, proceeded immediately to the operation. As ſhe had not made water for a conſiderable time, I endeavoured, after putting her in a proper poſition, to paſs the catheter; but the bladder was turned ſo much over the pubes, by the belly being exceedingly pendulous, that neither I nor my ſon could ſucceed, though we repeatedly tried both with a ſmall flat catheter and with a flexible one, aſſiſted by a finger in the vagina: however, in raiſing the belly a little, during theſe trials, about half a pint of water was diſcharged. I then performed the operation nearly in the manner deſcribed by M. Le Roy; but found great difficulty in making the upper part of the inciſion without wounding the bladder, from the above-mentioned circumſtance of the pendulous belly, which appeared juſt as if a ſmall ſugar loaf had been placed within the parietes of the abdomen, the baſe reſting upon the vertebræ lumborum, and the apex puſhing out below the navel.

 There

There being no convenient table in the houſe, I doubled the bolſters at the edge of the bed, (in order to gain every advantage of poſture I was able) and placed the patient with her hips as high and her ſhoulders as low as ſhe could bear them ; a woman ſitting at the ſame time, on each ſide, in a low chair, with one of the patient's feet in her lap.

To avoid wounding the bladder, after juſt dividing the integuments, I introduced the fore finger of my left hand, and kept it conſtantly behind the back of my knife, (a common diſſecting knife) and cut from within outwards, till I had divided the ſymphyſis. I had before cautioned the women who held the patient's feet to be careful not to pull her knees wider, but to keep them juſt as they were placed before the operation, and was much aſtoniſhed to find the firſt pain after the diviſion bring down the nates of the child to the os externum ; the next pain delivered the body ; and by giving the head the proper turns, I extracted it, though a very large one, with as much eaſe as any one I ever delivered.

The placenta having immediately followed, the wound was dreſſed, and the patient's knees were brought together and ſecured by a bandage ; dage; I could not bring them down, by reaſon of the contraction before mentioned; but a broad bandage being applied round the hips, ſhe was put to bed.

The child, notwithſtanding the patient's aſſertion of her feeling it, had been dead ſome days, and was quite putrid, the cuticle ſtripping off from every part.

The patient took an anodyne draught after the operation, and paſſed a comfortable night. She made water into a baſon ſeveral times, and I had ſome hopes of her recovery, though her pulſe ſtill continued much too quick. She frequently declared to her attendants that ſhe had ſuffered more in any two pains the preceding day than during the whole of the operation. She continued pretty eaſy all the next day (Thurſday), except that there was a little pain in her left ſide, which ſhe told me ſhe had felt ſome days before ſhe was taken in labour. Upon further inquiry I found it was the ſide of her belly that was painful, and perceived the uterus much too large, and painful when preſſed. Proper applications to the part affected, and ſaline medicines internally, were ordered, a clyſter was adminiſtered, and at night the anodyne draught was repeated.

She had a tolerable night, and the next morning I found her much better; but you will judge of my furprife and concern when, returning about four o'clock in the afternoon from vifiting fome patients out of town, I found her fitting at the table drinking tea, in a ftrong current of air betwixt the door and window, both of which were open, and with a large chafing difh of coals by her fide to keep the tea kettle boiling. I chid the nurfe feverely, ordered the patient immediately to bed, and enjoined the ftricteft repofe for the future.

On Saturday morning fhe was evidently worfe, had a troublefome cough, and the pain in her belly, which on Friday had almoft left her, was now much increafed. As the clyfter had done but little, a lenient mixture of tamarinds and foluble tartar was given, which operated very well, and in the evening I thought her rather better; but on Sunday morning fhe had a violent rigor, fucceeded by a burning fever, the pain in her belly was much increafed, and her pulfe was at 130, but extremely weak. On Monday the abdomen was much fwelled, a profufe fweat came on, which towards evening grew cold, and early on Tuefday morning fhe expired.

I have

I have been the more particular in relating thefe fubfequent fymptoms, as I think there is not the leaft reafon to fuppofe, from an impartial review of them, that her death was a confequence of the operation.

I could not get leave to examine the body till Thurfday, by which time it was putrid to a higher degree than I ever yet faw one, however long kept. Indeed my fon had a very difagreeable fever, attended with naufea and violent head-ach, from a fmall fcratch which he got on his finger in opening it. From fo putrid a fubject little knowledge was to be obtained, except the dimenfions of the pelvis; there were, however, very evident figns of inflammation upon the left fide of the fundus uteri. My fon had the addrefs to get the pelvis, which, when entirely freed from its contents, meafured from fide to fide $4\frac{1}{2}$ inches; from the pubis to the facrum $2\frac{1}{4}$ inches, juft at the fymphyfis, but jutting in on each fide to about an inch; and the fpace betwixt the tuberofities of the ifchia was but $1\frac{1}{2}$ inch.

The diameter of the child's head, with the integuments on, from the os frontis to the occiput, was $4\frac{1}{2}$ inches, from fide to fide 4 inches, both both meafures accurately taken with a pair of callipers.

There were two particulars which at the time were matters of aftonifhment to me, and I fhall juft therefore beg leave to make a few obfervations on them.

The firft was the extreme narrownefs of the pelvis, efpecially the fpace betwixt the tuberofities of the ifchia, which, when I delivered her fix years before, was not much lefs than the common fize. I cannot fuppofe her conftant fitting could alone produce that effect, as many people in particular trades fit nearly as much as fhe did. The other was the furprifing eafe with which fhe was delivered after the divifion took place. My fon foon difcovered the reafon of both; for in diffecting the pelvis, for drying, he found the bones fo foft, that he could run his knife into moft parts of them with the greateft eafe. I cannot find that her way of living was in any refpect different from that of other poor people, and therefore cannot account for this uncommon foftnefs of the bones. Perhaps this very circumftance may be an objection with fome to the performing the operation in future, as we cannot expect fo great great a ſeparation of the parts in a ſubject where ſuch ſoftneſs does not prevail. But when we conſider the extraordinary diſproportion betwixt the ſize of this pelvis and that of the head, and recollect that the woman neither complained of pain in the divided parts, nor in her back, from any diſtention of the poſterior ligaments, but was even able to ſit at the tea table the ſecond day after the operation, I think in moſt caſes there is great reaſon to expect a ſucceſsful event, more eſpecially if M. Le Roy's aſſertion be true, that not only the cartilages but the bones of the pelvis become ſofter during the laſt months of pregnancy, from " a ſolution of the earthy matter in the " mother, which is converted to the uſe of " the fœtus." Every one's experience evinces that the callus in fractures forms more ſlowly during pregnancy, and in ſome caſes not at all till ſome time after delivery.

It may poſſibly be aſked why the operation was not propoſed ſooner? I have only to anſwer, that both myſelf and my ſon, perceiving a conſiderable motion in the bones of the pelvis during every ſtrong pain, I was willing to hope if the child had been ſmall, it might have been delivered delivered without it: and I cannot help recommending a large degree of patience to the younger part of the profeſſion eſpecially, having, in an extenſive practice of more than thirty years, ſeen the good effects of ſuch caution, and having had the happineſs more than once of reprieving children condemned to the crotchet, who were afterwards delivered with ſafety to themſelves and the mother.

